# p53 and TDG are dominant in regulating the activity of the human *de novo* DNA methyltransferase DNMT3A on nucleosomes

**DOI:** 10.1074/jbc.RA120.016125

**Published:** 2020-11-24

**Authors:** Jonathan E. Sandoval, Norbert O. Reich

**Affiliations:** 1 Department of Molecular, Cellular and Developmental Biology, University of California, Santa Barbara, California, USA; 2 Department of Chemistry and Biochemistry, University of California, Santa Barbara, California, USA

**Keywords:** DNA methyltransferase 3A (DNMT3A), DNA methylation, p53, TDG, histone modifications, H3K4me0, H3K4me3, protein–protein interactions, epigenetics, AML, AML, acute myeloid leukemia, DNMT3A, DNA methyltransferase 3A, TDG, thymine DNA glycosylase

## Abstract

DNA methylation and histone tail modifications are interrelated mechanisms involved in a wide range of biological processes, and disruption of this crosstalk is linked to diseases such as acute myeloid leukemia. In addition, DNA methyltransferase 3A (DNMT3A) activity is modulated by several regulatory proteins, including p53 and thymine DNA glycosylase (TDG). However, the relative role of histone tails and regulatory proteins in the simultaneous coordination of DNMT3A activity remains obscure. We observed that DNMT3A binds H3 tails and p53 or TDG at distinct allosteric sites to form DNMT3A–H3 tail-p53 or –TDG multiprotein complexes. Functional characterization of DNMT3A–H3 tail-p53 or –TDG complexes on human-derived synthetic histone H3 tails, mononucleosomes, or polynucleosomes shows p53 and TDG play dominant roles in the modulation of DNMT3A activity. Intriguingly, this dominance occurs even when DNMT3A is actively methylating nucleosome substrates. The activity of histone modifiers is influenced by their ability to sense modifications on histone tails within the same nucleosome or histone tails on neighboring nucleosomes. In contrast, we show here that DNMT3A acts on DNA within a single nucleosome, on nucleosomal DNA within adjacent nucleosomes, and DNA not associated with the DNMT3A–nucleosome complex. Our findings have direct bearing on how the histone code drives changes in DNA methylation and highlight the complex interplay between histone tails, epigenetic enzymes, and modulators of enzymatic activity.

Carried out by DNA methyltransferase 3A (DNMT3A), *de novo* 5-methylcytosine patterning of mammalian DNA is a major epigenetic modification frequently associated with transcriptional repression (1, 2). The plethora of posttranslational modifications to specific residues within the amino-terminal tails of core histones (H2A, H2B, H3, and H4) forms another epigenetic process leading to the activation or repression of genes (3). Mammalian transcriptional regulation relies on the extensive crosstalk between histone modifications and DNA methylation, and changes in this interplay are a major contributor to human cancers. For example, genome-wide epigenetic profiling reveals that genomic loci with H3K36me2/3, H3K9me3, and H3K4me0 correlate with enrichment of *de novo* DNA methylation (4, 5, 6); more specifically, DNMT3A-mediated methylation follows H3K9me3 and H3K36me3 patterning (7, 8). Furthermore, alterations to the interplay between DNA methylation and modifications to H3K4/K27 contribute to the altered expression of the *cyclin-dependent kinase inhibitor 2B* (*CDKN2B* or *p15*) gene observed in acute myeloid leukemia (AML) (9). However, the mechanisms that underpin these correlations between changes in histone modifications and DNA methylation remain obscure, as is the contribution of regulatory proteins in this context. We envision two plausible situations (Fig. 1*B*), starting with the physical recruitment of DNMT3A (Fig. 1*B*, I.) or DNMT3A in complex with distinct regulatory proteins (Fig. 1*B*, II. and V.), through its well-known interactions with histone tails. In this model, DNMT3A acts as a reader of histone marks (Fig. 1*B*, I., II., and V.) with histone tails modulating enzymatic activity (Fig. 1*B*, I., II., and V.) or alternatively with histone tails primarily recruiting DNMT3A and regulatory proteins playing a dominant role in the modulation of enzymatic activity (Fig. 1*B*, II. and V.). An additional scenario derives not from a physical association of DNMT3A and particular histone marks but rather the regulatory proteins associated with DNMT3A serving as a reader of histone marks (Fig. 1*B*, VI.), and the enzymatic activity of DNMT3A modulated by regulatory proteins or a combination of regulatory protein–histone tail interactions (Fig. 1*B*, VI.). Clearly, these mechanisms are not mutually distinctive.

**Figure 1:**
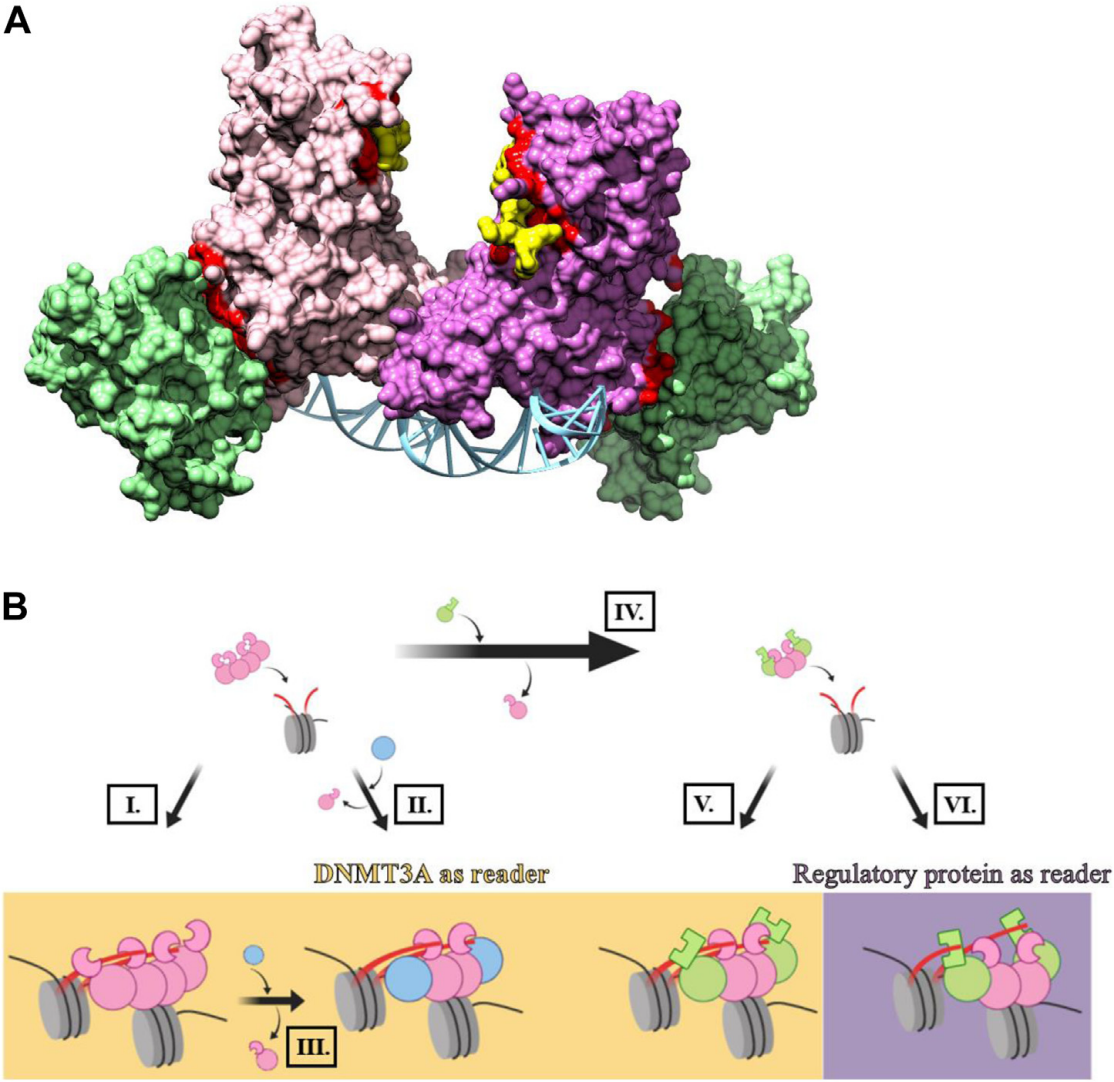
**DNMT3L and H3 tails bind distinct surfaces on DNMT3A for modulation of enzymatic activity.***A*, surface model of a DNMT3A heterotetramer ( and ) (residues 468–912) bound by DNMT3L () (residues 171–379) and Histone H3 N-terminal peptide () (residues 1–12) (adapted from PDB 4U7T) (10). The DNA (*blue*) binding interface on PDB 4U7T was modeled based on the structural similarity to a DNA-bound DNMT3A-DNMT3L crystal structure (PDB 5YX2) (50). () denote interacting surfaces on DNMT3A for DNMT3L or H3 peptide interactions (<5 Å). *B*, depiction of proposed interactions associated with the targeting of DNMT3A homotetramers (I.) or heterotetramers (II.–IV.) to nucleosome substrates. The *yellow panel* encompasses complexes in which DNMT3A is acting as the reader of histone marks (I., II., and V.), whereas the purple panel represents complexes in which regulatory proteins associated with DNMT3A serve as the reader of histone marks (VI.). *Pink* represents DNMT3A, *blue* denotes regulatory proteins that lack a histone reading domain, and *green* represents regulatory proteins of DNMT3A that additionally act as readers of histone marks. DNMT3A, DNA methyltransferase 3A; DNMT3L, DNA methyltransferase 3 like.

The crystal structure of a DNMT3A–DNA Methyltransferase 3 Like (DNMT3L) heterotetramer in complex with a histone H3 peptide (residues 1–21) reveals that DNMT3A binds histone tails *via* its conserved ATRX-DNMT3-DNMT3L (ADD) domain while simultaneously accommodating DNMT3L at the tetramer interface (Fig. 1*A*) (10). To date, insights on the combinatorial effect of regulatory proteins and histone H3 tails in the modulation of DNMT3A activity have focused solely on DNMT3L, which is complicated by the fact that both DNMT3A and DNMT3L bind H3 tails *via* the ADD domain (Fig. 1*B*, V. and VI.) (10, 11, 12). The interactions between DNMT3A and nucleosomes and regulatory proteins like DNMT3L is further modulated by other regulatory proteins such as tumor suppressor p53 (p53) or thymine DNA glycosylase (TDG), which involve DNMT3A surface regions that are shared with DNMT3L (13, 14). While there is no evidence for the direct interactions of p53 or TDG with histone tails, their genomic locations are associated with specific histone modifications and indirectly mediating changes to the modifications of histone tails (15, 16). Thus, these proteins clearly contribute to the modulation of epigenetic mechanisms. Although the related interactions between DNMT3A and nucleosomes remain largely uncharacterized, several studies have investigated this relationship in histone modifying enzymes. For example, the chromodomain helicase DNA-binding protein 4 associates with histone H3 tails within a single nucleosome (intranucleosomal interactions), whereas heterochromatin protein 1α binds individual histone H3 tails in adjacent nucleosomes (internucleosomal interactions) (17, 18). The relationship between DNMT3A and histone H3 tails is inherently intricate as DNMT3A plays a dual role as a reader of histone H3 tails and a writer on nucleosomal DNA (10). Furthermore, when the possible combinations of biologically significant complexes involving regulatory proteins are considered (Fig. 1*B*), the complexity of the dynamics associated with epigenetic control is evident. Our current understanding of the allosteric modulation of DNMT3A activity is limited to studies focusing on the individual roles of histone H3 tails, regulatory proteins, or the interplay between DNMT3L and histone H3 tails (7, 10, 11, 12, 13, 14, 19).

Our interest here is to explore the relative role (dominant or passive) of histone H3 tails and regulatory proteins in the modulation of DNMT3A activity to better understand the potential crosstalk between histone H3 tails and regulatory proteins and how this translates into meaningful biological outcomes. Our approaches rely on human-derived synthetic histone H3 tails, mononucleosomes or polynucleosomes, the regulatory proteins p53 and TDG, and a modified pulse–chase assay along with florescence anisotropy assays. Our work provides novel insights into the dynamic interplay between distinct epigenetic mechanisms as well as a better understanding of the regulation of enzyme activity in protein complexes consisting of modulators that bind distinct allosteric sites.

## Results

### Regulation of full-length DNMT3A activity by p53 or TDG is dominant in DNMT3A–p53–H3 tail or DNMT3A–TDG–H3 tail complexes

DNMT3A simultaneously accommodates DNMT3L and histone H3 tails through interactions at distinct surfaces (Fig. 1*A*) (10). In conjunction with a distinct co-crystal structure of DNMT3L bound to H3K4me0 peptide (PDB 2PVC) (11), functional studies of the interactions between DNMT3A, DNMT3L, and H3 peptide have led to a model in which recognition of H3K4me0 by DNMT3L leads to the recruitment of DNMT3A (10, 11, 12). However, this model does not entirely explain the relationship between DNMT3L and histone H3 tails in the simultaneous regulation of DNMT3A activity in DNMT3A–DNMT3L–H3 complexes as the same activation is observed in the absence of DNMT3L (19). Furthermore, this model leaves unanswered whether histone tails primarily recruit DNMT3A, while DNMT3L plays a dominant role in the modulation of DNMT3A activity (Fig. 1*B*, V.), or DNMT3L primarily recruits DNMT3A, and the activity of DNMT3A is modulated by DNMT3L or a combination of DNMT3L–histone H3 peptide interactions (Fig. 1*B*, VI.). To elucidate the role of histone H3 tails and regulatory proteins in the coordination of DNMT3A activity, we assessed the dynamics and functional consequences of complexes involving DNMT3A, histone H3 peptides (H3K4me0 and H3K4me3) and two previously characterized regulatory proteins of DNMT3A (p53 or TDG) whose cellular functions have been associated with the presence of specific histone modifications (13, 14, 15, 16).

**Figure 2:**
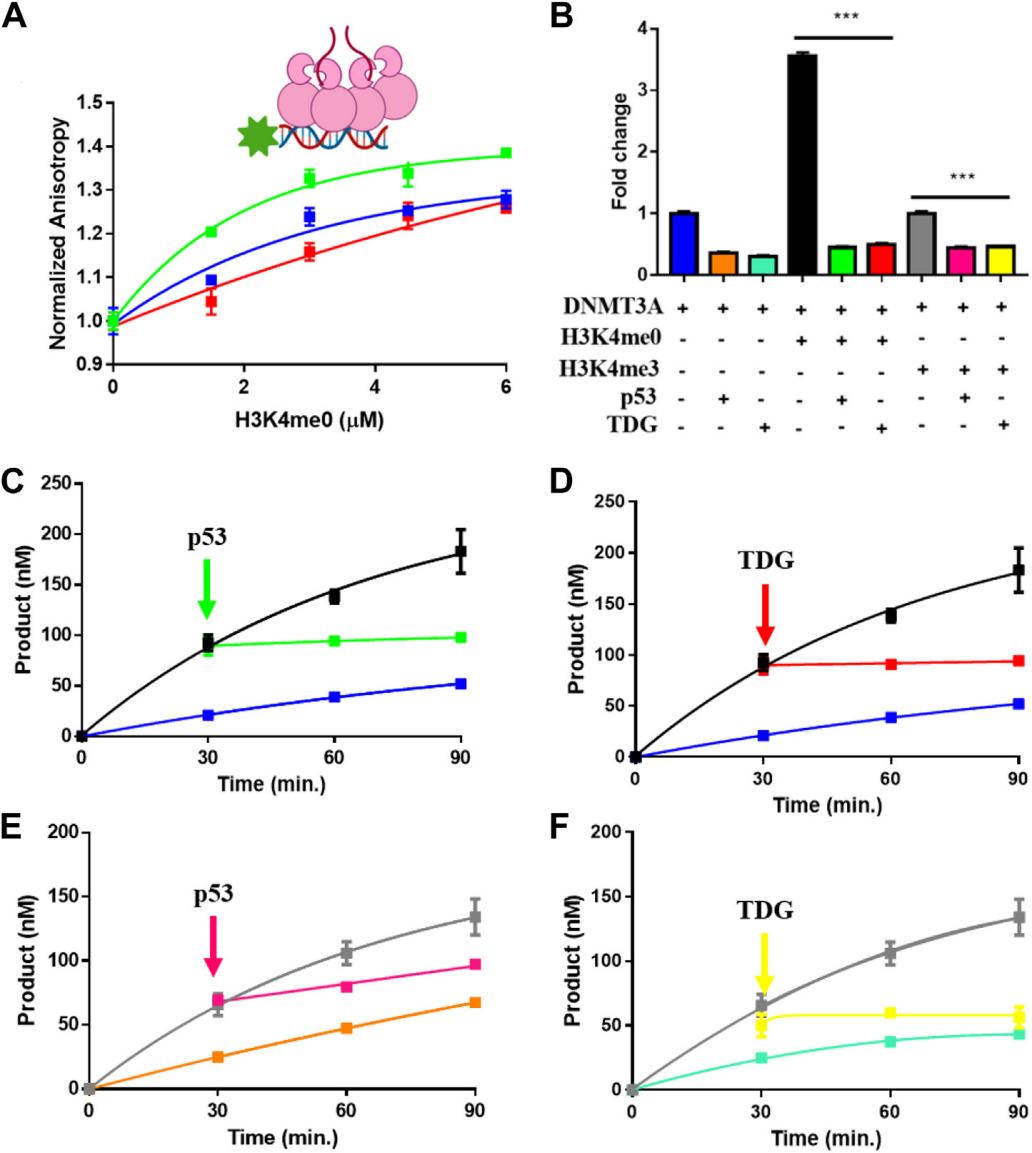
**Modulation of DNMT3A activity by regulatory proteins is dominant in the presence of H3K4me0 or H3K4me3 peptides.***A*, the addition of H3K4me0 peptide (unlabeled) increases the FA of DNA-bound DNMT3A (), DNMT3A–p53 (), or DNMT3A–TDG () complexes. In (*A*), 50 nM DNA (5′ 6-FAM-labeled GCbox30; see methods for sequence) was preincubated with DNMT3A or DNMT3A with individual regulatory proteins (1 μM at 1:1 to DNMT3A tetramer). Data (*A*) are normalized to FA values in the absence of H3K4me0. *B*, the presence of H3K4me0 or H3K4me3 does not disrupt inhibition of DNMT3A enzymatic activity by p53 or TDG in equilibrium reactions. In (*B*), data were normalized to the DNA methylation activity observed in DNMT3A on poly dI-dC as a substrate and are representative of reactions carried out for 1 h. The addition of p53 (*C*, *E*) or TDG (*D*, *F*) disrupts actively catalyzing DNMT3A–H3K4me0 (*C*–*D*) or DNMT3A–H3K4me3 (*E* and *F*) complexes. Reactions consisting of DNMT3A only (*C*–*D*), DNMT3A–p53 (*B* and *E*) or -TDG (*B* and *E*) co-incubations were performed as controls. For co-incubations, proteins were placed at 37 °C for 1 h before the addition of substrate DNA. Except for (*E*) and (*F*) with proteins at 50 nM (1:1 to DNMT3A tetramer), DNA methylation reactions consisted of proteins at 150 nM (1:1 to DNMT3A tetramer), H3K4 peptides were at 4 μM and were initiated by the addition of 5 μM bp poly dI-dC. Data reflect the mean ± S.D. of three experiments; (*B*) one-way analysis of variance was used to compare the values of p53 or TDG with DNMT3A to similar reactions but in the presence of H3K4me0 or H3K4me3; ∗∗∗*p* < 0.001; ns, *p* > 0.05. DNMT3A, DNA methyltransferase 3A; TDG, thymine DNA glycosylase.

We previously used fluorescence anisotropy to characterize the interactions between the catalytic domain of DNMT3A and p53 on a fluorophore-labeled oligonucleotide (5′ 6-FAM-labeled duplex DNA [GCbox30]) containing a single recognition site for DNMT3A (14). We relied on this approach to assess the dynamics between H3K4me0 and DNMT3A–p53 or DNMT3A TDG on DNA. Importantly, the modulation of DNMT3A activity on DNA by p53 and TDG does not occur with other DNA cytosine methyltransferases, which argues against these effects deriving from competition by these regulatory proteins resulting from DNA binding (13, 14). Consistent with previous findings (10), increasing concentrations of unlabeled H3K4me0 peptide to a fixed concentration of DNA-bound DNMT3A (Fig. 2*A* ) increases the fluorescence anisotropy signal, reflecting the formation of DNA-bound DNMT3A–H3K4me0 complexes. Similarly, preformed DNMT3A–p53 (Fig. 2*A* ) or DNMT3A–TDG (Fig. 2*A* ) complexes on DNA displayed an increase to the initial anisotropy signal with a corresponding increase in H3K4me0 peptide concentration, suggesting the formation of higher order DNMT3A heterotetramers in complex with H3K4me0 peptide on DNA. Given that DNA-bound DNMT3A heterotetramers with p53 (Fig. 2*A* ) or TDG (Fig. 2*A* ) can accommodate H3K4me0 peptide, we sought to assess the relative role of H3K4 and regulatory proteins (p53 or TDG) in the simultaneous modulation of DNMT3A enzymatic activity.

As previously observed (19), preincubation of DNMT3A with H3K4me0 peptide results in activation of enzymatic activity (Fig. 2*B* ), whereas preincubation of DNMT3A with H3K4me3 peptide (Fig. 2*A* ) results in comparable levels of activity as reactions without H3 peptides (Fig. 2*B* ), although DNMT3A binds both peptides *in vitro* (10). Additionally, we observed a roughly 50% decrease in DNMT3A activity in control reactions consisting of DNMT3A–p53 (Fig. 2*B* ) or DNMT3A–TDG (Fig. 2*B* ) preincubations as previously reported (13, 20). In equilibrium reactions where DNMT3A and H3K4me0 peptide were preincubated with individual regulatory proteins, we observed that inhibition of DNMT3A activity by p53 (Fig. 2*B* ) or TDG (Fig. 2*B* ) is dominant over H3K4me0 peptide activation of DNMT3A (Fig. 2*A* ). Under similar experimental conditions, the presence of H3K4me3 peptide does not disrupt modulation of DNMT3A methylation activity by p53 (Fig. 2*B* ) or TDG (Fig. 2*B* ). In fact, preincubation of DNMT3A with p53 or TDG in the presence of H3K4me0 or H3K4me3 peptides led to comparable levels of DNMT3A-dependent methylation as reactions consisting of DNMT3A with only p53 (Fig. 2*B* ) or TDG (Fig. 2*B* ). To further challenge the dominant modulatory effect of p53 or TDG over H3K4me0 or H3K4me0 peptides on DNMT3A observed, we then evaluated the functional outcome of adding equimolar concentrations of individual regulatory proteins (ratio of 1:1 regulatory protein to 150 nM tetramer DNMT3A) to DNMT3A–H3K4me0 or DNMT3A–H3K4me3 complexes that are actively methylating DNA. Like reactions at equilibrium (Fig. 2*B*), the addition of p53 (Fig. 2*C* ) or TDG (Fig. 2*D* ) to actively methylating DNMT3A–H3K4me0 complexes disrupted H3K4me0 peptide-mediated stimulation of DNMT3A activity (Fig. 2, *C*–*D* ). Furthermore, actively catalyzing DNMT3A–H3K4me3 peptide complexes (Fig. 2, *E*–*F* ) are responsive to the addition of p53 (Fig. 2*E* ) or TDG (Fig. 2*F* ). While H3K4me0 is associated with gene silencing, trimethylated histone H3K4 (H3K4me3) sites are associated with active gene promoters (21). These results do not derive from direct p53 or TDG competition with DNMT3A binding to the DNA (Fig. S6) (13, 14). Our results indicate that in DNMT3A–H3 tail-regulatory protein complexes (Fig. 2*A*), regulatory proteins play a dominant role in the simultaneous coordination of DNMT3A activity despite the methylation state H3K4 (Fig. 2, *B*–*F*).

### Full-length DNMT3A methylates internucleosomal DNA

**Figure 3:**
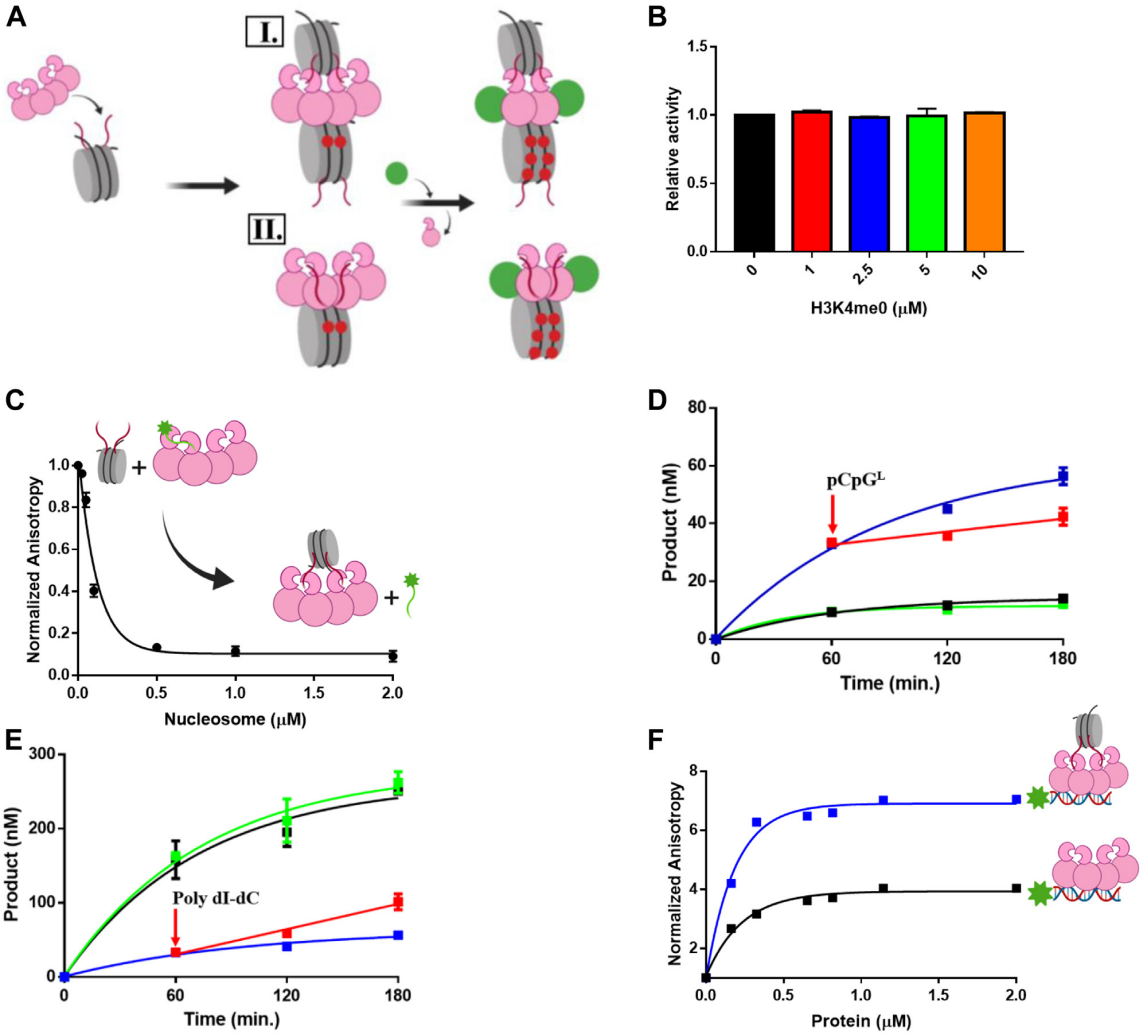
**DNMT3A remains bound to histone tails in mononucleosomes when acting on free DNA.***A*, proposed interactions for DNMT3A, or DNMT3A in complex with regulatory proteins, with nucleosomes: I. internucleosomal and II. Intranucleosomal. *B*, increasing the concentration of H3K4me0 peptide does not disrupt the enzymatic activity of DNMT3A in equilibrium reactions. In (*B*), data were normalized to the DNA methylation activity of DNMT3A in the absence of H3K4me0 and are representative of reactions carried out for 1 h (*C*). The addition of unlabeled mononucleosomes disrupts DNMT3A (150 nM tetramer) bound to FAM-labeled H3K4me0 (2 μM); data are normalized to FA values in the absence of mononucleosomes. Catalytically active DNMT3A on mononucleosomal DNA as a substrate (*D*–*E*) is responsive to the addition of excess (20X) endogenous pCpG^L^ (*D*) or Poly dI-dC (*E*). In (*D*–*E*), reactions consisted of DNMT3A at 150 nM tetramer and mononucleosomes at 1 μM (*B*–*E*), and the following reactions were also performed as controls: pCpG^L^ only (*D* ), mixture of pCpG^L^ and mononucleosomes (*D*), Poly dI-dC only (*E*) and mixture of Poly dI-dC and mononucleosomes (*E*). Increasing concentrations of preformed DNMT3A–mononucleosome complexes to FAM-labeled DNA (15 nM; see methods for sequence) (*F*) led to a greater change in FA relative to similar binding reactions in the absence of mononucleosomes (*F*). Data reflect the results of two independent experiments. DNMT3A, DNA methyltransferase 3A.

Elucidating the spatial relationship between epigenetic enzymes and their substrates (intranucleosomal or internucleosomal action, Fig. 3*A*) is essential to truly understand nucleosome-protein interactions and the structural basis of epigenetic gene regulation. Several studies have characterized this relationship in the context of histone-modifying enzymes (17, 18). However, the molecular arrangement between DNMT3A and nucleosome substrates remains less well characterized (Fig. 3*A*). The primary focus has been on whether the enzyme methylates linker DNA or the DNA wrapped to form the nucleosome, which at this point remains unclear. To provide insights into the orientation of DNMT3A relative to nucleosome substrates, we assessed the accessibility of exogenous (non-nucleosomal substrate) H3K4me0 peptide or DNA to DNMT3A-mononucleosome complexes (Fig. 3, *B*–*F*). We initially assessed whether the N-terminus of DNMT3A contributes to DNMT3A–mononucleosome interactions. Consistent with previous findings, the catalytic domain of DNMT3A (Δ1–611) (Fig. S1, ) and the prokaryotic CpG DNA methyltransferase M. SssI (Fig. S1, ) displayed reduced activity on unmodified mononucleosomal substrates relative to full-length DNMT3A (Fig. S1, ) (12, 22). We then assessed the effect of increasing concentrations of H3K4me0 peptide on the activity of DNMT3A using mononucleosomes as a substrate (Fig. 3*B*). Unlike reactions consisting of free DNA (Fig. 2), an increase in the concentration of H3K4me0 peptide did not alter the enzymatic activity of DNMT3A on mononucleosomal DNA (Fig. 3*B*). Thus, the association of DNMT3A to intrinsic (mononucleosomal) H3 tails appears to perturb the activation of DNMT3A by H3K4me0 peptide. To additionally challenge this notion, we then monitored changes in the fluorescence anisotropy of FAM-labeled H3K4me0 (1, 2, 3, 4, 5, 6, 7, 8, 9, 10, 11, 12, 13, 14, 15, 16, 17, 18, 19, 20, 21)–DNMT3A complexes by the addition unlabeled mononucleosomes (Fig. 3*C*). The addition of DNMT3A (150 nM tetramers) to 2 μM FAM-labeled H3K4me0 peptide leads to saturating anisotropy (Fig. S5). Under these conditions, we observed that the addition of unlabeled mononucleosomes (0–2 μM) led to a robust decrease to the initial fluorescence anisotropy of FAM-labeled H3K4me0 peptide bound by DNMT3A (Fig. 3*C*). The results obtained are consistent with H3 tails in mononucleosomes displacing FAM–H3K4me0 peptide in DNMT3A–H3K4me0 peptide complexes.

To evaluate the accessibility of extrinsic DNA (non-nucleosome) to DNMT3A, we monitored DNMT3A-mediated methylation following the addition of a 20-fold excess of pCpG^L^ (a plasmid with ∼3800 base pairs and no CpG sites) or Poly dI-dC (a synthetic DNA substrate with ∼2000 base pairs and 800 CpG sites) to DNMT3A acting on mononucleosomal DNA (1 μM) (Fig. 3, *D*–*E*). The robust activity of DNMT3A on Poly dI-dC and poor activity on pCpG^L^ allow for effective monitoring of any changes to DNMT3A activity on mononucleosomal DNA. Initial controls were performed in which reactions were initiated by the addition of a mixture of excess (20-fold) pCpG^L^ to mononucleosomes (Fig. 3*D* ) or a mixture of excess (20-fold) Poly dI-dC to mononucleosomes (Fig. 3*E* ). We found that reactions initiated by the addition of a mixture of pCpG^L^ and mononucleosomes (Fig. 3*D* ) or Poly dI-dC and mononucleosomes (Fig. 3*E* ) resulted in comparable levels of activity as reactions initiated by the addition of only pCpG^L^ (Fig. 3*D* ) or Poly dI-dC (Fig. 3*E* ). The addition of pCpG^L^ (Fig. 3*D* ) or Poly dI-dC (Fig. 3*E* ) 60 min into the reaction resulted in a respective decrease (Fig. 3*D* ) or increase (Fig. 3*E* ) to the activity of DNMT3A catalyzing on mononucleosomal DNA (Fig. 3, *D*–*E* ). Thus, DNMT3A appears to act on extrinsic DNA (pCpG^L^ or Poly dI-dC) while bound and acting on mononucleosomes. We then challenged this notion by tracking the fluorescence anisotropy of FAM-labeled Gcbox30 DNA after the addition of preformed DNMT3A–mononucleosome to assess the ability of DNMT3A–mononucleosome complexes to bind non-nucleosomal DNA (Gcbox30). An increase in the concentration of preformed DNMT3A–mononucleosome complexes (Fig. 3*F* ) led to a greater increase to the initial fluorescence anisotropy of FAM-labeled Gcbox30 DNA relative to the addition of DNMT3A only (Fig. 3*F* ), indicating the formation of a higher order complex composed of preformed DNMT3A–mononucleosome complexes bound to DNA (Gcbox30). Thus, our combined results are most consistent with an internucleosomal mechanism (Fig. 3*A*, I.) in which DNMT3A that is already bound to a nucleosome can act on another DNA molecule, which is not part of the initial DNMT3A–nucleosome complex.

### Modulation of full-length DNMT3A activity by p53 or TDG is not impeded by nucleosomes

**Figure 4:**
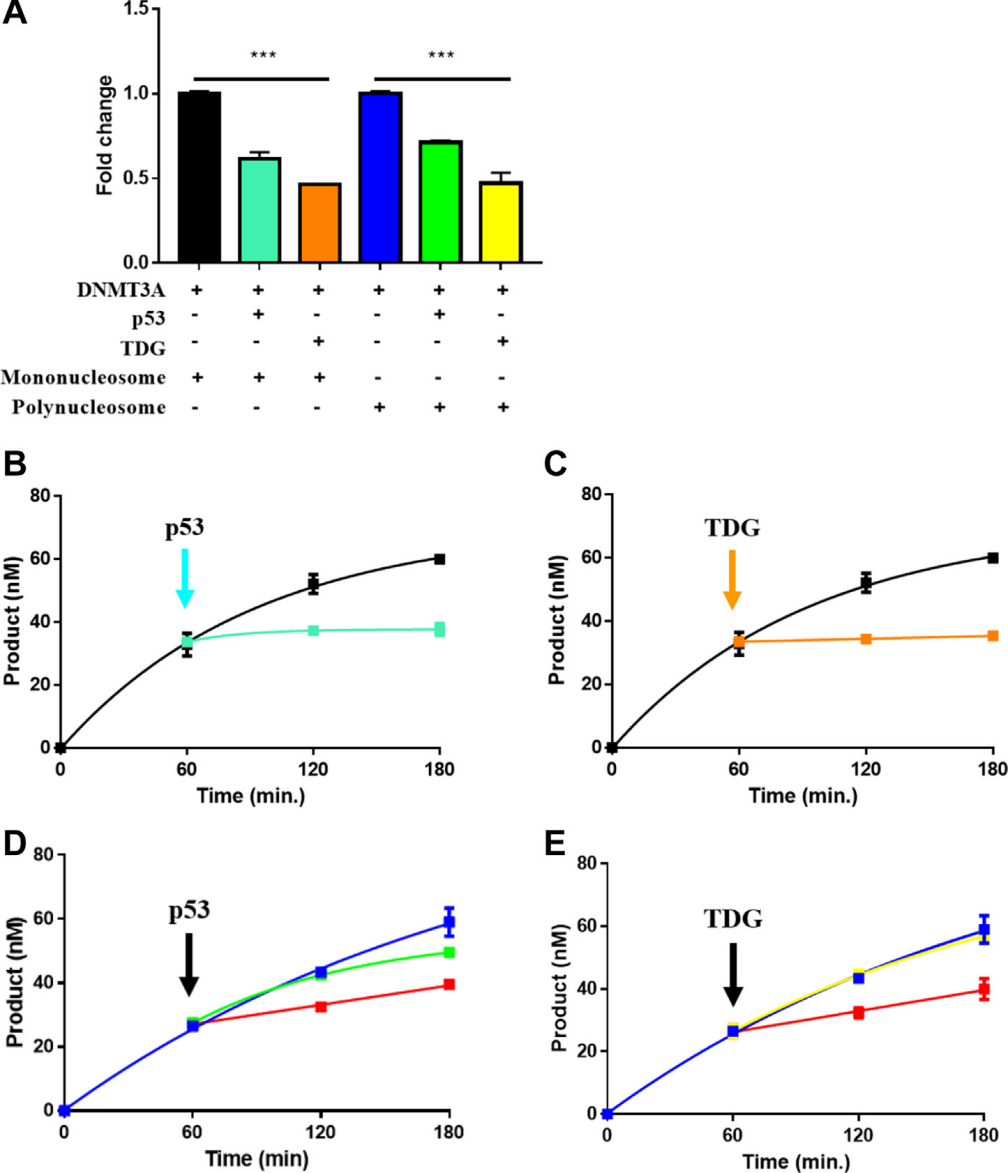
**Modulation of DNMT3A activity by p53 or TDG using human mononucleosomes or polynucleosomes.***A*, modulation of DNMT3A activity by p53 or TDG is unaffected in equilibrium reactions with mononucleosomal or polynucleosomal DNA as a substrate (1 μM). The modulatory effect on DNMT3A activity observed in equilibrium reactions (*A*) persists in actively catalyzing DNMT3A on mononucleosomal DNA (*B*–*C*) challenged by the addition of p53 (*B* ) or TDG (*C*). The addition of p53 or TDG disrupts actively catalyzing DNMT3A on polynucleosomal DNA at excess concentrations p53 (*D*) or TDG (*E* ) but not at equimolar amounts p53 (*D*) or TDG (*E*). DNA methylation reactions consisted of proteins at 150 nM (1:1–150 nM tetramer DNMT3A) or excess regulatory proteins at 500 nM (see methods) (*D* and *E*). In (*A*), reactions consisting of mononucleosomes or polynucleosomes (1 μM) and individual regulatory proteins (p53 or TDG, 1:1 at 150 nM) were initiated by the addition of DNMT3A. In (*B*), actively catalyzing DNMT3A with mononucleosomes or polynucleosomes (1 μM) as a substrate was challenged by the addition of p53 or TDG (1:1 at 150 nM) after 1 h. Data from reactions in (*A*) were normalized to the DNA methylation activity observed in DNMT3A on mononucleosomes or polynucleosomes and are representative of reactions carried out for 1 h. Data reflect the mean ± S.D. of three experiments; (*A*) one-way analysis of variance was used to compare the values of p53 or TDG to those of DNMT3A for each substrate; ∗∗∗, *p* < 0.001; ns, *p* > 0.05. DNMT3A, DNA methyltransferase 3A; TDG, thymine DNA glycosylase.

Studies of the enhancer of zeste 2 polycomb repressive complex 2 subunit histone H3 methyltransferase reveal that enhancer of zeste 2 polycomb repressive complex 2 subunit exhibits a 5-fold increase in histone methylation activity on polynucleosomes (>10) relative to mononucleosomes (23). MNase digestion of native chromatin isolated from mouse embryonic stem cell nuclei shows that DNMT3A primarily binds mononucleosomes or polynucleosomes (<7) and higher-order polynucleosomes (>12) to a lesser extent (24). Based on these observations and previous work on p53 and TDG along with their links to the regulation of distinct epigenetic mechanisms, we assessed the catalytic activity of DNMT3A as well as the ability of p53 or TDG to modulate DNMT3A using mononucleosome or polynucleosome substrates (Fig. 4) (13, 14, 15, 16). We initially relied on DNA methylation assays across a range of mononucleosome or polynucleosome concentrations to generate saturation curves for DNMT3A on each substrate (Fig. S2). DNMT3A displayed an increased *K*_M_ on polynucleosome (336 ± 51 nM; Fig. S2 ) relative to mononucleosomes (86.9 ± 14 nM; Fig. S2, ). DNMT3A had comparable maximal velocity (approximately 0.8 nM product/min) at saturating mononucleosome or polynucleosome concentrations (Fig. S2). Thus, DNMT3A requires saturating polynucleosome concentrations to overcome the hindered accessibility to DNA, which likely stems from the structural complexity of polynucleosomes.

DNMT3A moves along DNA substrates carrying out multiple cycles of methylation on the same piece of DNA before dissociating, and the DNA-bound DNMT3A is accessible for modulation by distinct regulatory proteins (13, 14, 25, 26). Given that DNMT3A acts on nucleosomal DNA without dissociating from histone N-Terminal tails (Fig. 3, *B*–*F*; Fig. S1), we assessed whether mononucleosomal or polynucleosomal DNA-bound DNMT3A is accessible for modulation by p53 or TDG (Fig. 4). In equilibrium reactions using mononucleosomal DNA, the presence of p53 (Fig. 4*A*, ) or TDG (Fig. 4*A*, ) results in decreased DNMT3A-mediated methylation relative to reactions consisting of DNMT3A only (Fig. 4*A*, ). Moreover, we observed that the modulatory effect on DNMT3A activity by p53 (Fig. 4*A*, ) or TDG (Fig. 4*A* ) observed in equilibrium reactions persisted in reactions with actively catalyzing DNMT3A on mononucleosomal DNA (B and C, ) challenged by the addition of p53 (Fig. 4*B* ) or TDG (Fig. 4*C* ) at equimolar concentrations relative to DNMT3A (150 nM tetramer). Therefore, the association of DNMT3A to histone tails while catalyzing mononucleosomal DNA does not occlude the accessibility of p53 or TDG to DNMT3A. Polynucleosomes challenge the ability of DNMT3A to access DNA compared with mononucleosomes (Fig. S2). Therefore, we also examined the ability of p53 or TDG to access and modulate DNMT3A activity on polynucleosome substrates. Like reactions with mononucleosomes (Fig. 4*A*), equimolar concentrations of p53 (Fig. 4*A* ), or TDG (Fig. 4*A* ) relative to DNMT3A (150 nM tetramer) inhibit DNMT3A acting on polynucleosomal DNA (Fig. 4*A* ) in equilibrium reactions. Surprisingly, the addition of equimolar amounts of p53 (Fig. 4*D* ) or TDG (Fig. 4*E* ) relative to DNMT3A (150 nM tetramer) methylating polymononucleosomal DNA (Fig. 4, *D*–*E* ) did not disrupt DNMT3A activity. Modulation of DNMT3A in transient reactions was only observed by the addition of excess (500 nM tetramer) p53 (Fig. 4*D* ) or TDG (500 nM dimer) (Fig. 4*E* ) compared with DNMT3A (150 nM tetramer). Our results show that although the structural complexity of polynucleosomes challenges the accessibility of p53 or TDG to actively catalyzing DNMT3A, p53 or TDG bind and modulate the enzymatic activity of DNMT3A in a concentration-dependent manner under catalytic conditions. In sum, our findings indicate that in DNMT3A-histone tail-regulatory protein (p53 or TDG) complexes, histone tails primarily sequester DNMT3A to nucleosomes, and p53 or TDG play a dominant role in the modulation of DNMT3A activity (Fig. 1*B*, II. and III.).

## Discussion

Studies aiming to characterize the mammalian epigenetic landscape show DNA methylation and histone tail modifications are highly interrelated mechanisms that regulate gene expression. Evidence of the crosstalk between DNA methylation and histone tail modifications include the positive correlation of DNA methylation with H3K36me2, H3K9me3, and H3K4me0 along with the altered expression of tumor-suppressor genes from changes to this crosstalk observed in AML (4, 5, 6, 7, 8, 9). While histone marks evidently provide cues for DNA methylations, the mechanisms underlying these correlations along with the role of regulatory proteins in the interplay between DNA methylation and histone marks remain unclear. The ADD domains of DNMT3A, DNMT3B, and DNMT3L bind H3K4me0 with comparable specificity while carrying out distinct biological functions, thereby suggesting ADD domain-H3 tail interactions are not entirely responsible for the individual differences in cellular activity (7, 8, 11, 12, 27, 28). Given that DNMT3A activity is modulated by a wide range of protein partners, interactions with regulatory proteins provide an additional mechanism to alter DNMT3A function (13, 14, 20, 29). Although studies of histone-modifying enzymes have included interactions with respect to nucleosomes (intranucleosomal or internucleosomal interactions; Fig. 3*A*), the mechanism of substrate engagement by DNMT3A remains obscure (17, 18). Based on this evidence and the previously characterized activities of p53 and TDG in the modulation of DNA methylation and histone tail modifications (13, 14, 15, 16), we sought to characterize the dynamics and simultaneous coordination of DNMT3A activity by p53 or TDG in the presence of H3 tails. Furthermore, we provide insights into the spatial relationship between DNMT3A and nucleosome substrates to better understand the interactions associated with DNMT3A acting as a reader of histone marks and how these interactions influence the modulation of its enzymatic activity. We show that modulation of DNMT3A methylation activity by p53 or TDG is dominant in the presence of histone H3 peptides or with the use of mononucleosome or polynucleosome substrates. Furthermore, we provide evidence for DNMT3A methylating internucleosomal DNA. Our findings provide insights into the intricate interactions of key epigenetic players and provide a molecular basis for how these interactions contribute to epigenetic transcriptional regulation.

Previous work from our lab has shown DNMT3L, p53, and TDG bind a common surface on DNMT3A (tetramer interface, Fig. 1*A*), which differs from the surface H3 tail binds on DNMT3A (ADD domain, Fig. 1*A*) (13, 14). Given that p53 and TDG lack a structural domain that directly associates with histone H3 tails, we propose that the crosstalk between p53 or TDG and histone H3 tails in the simultaneous modulation of DNMT3A activity (Fig. 1*B*, I.) is fundamentally different than DNMT3L in DNMT3A–DNMT3L–H3 tail complexes as DNMT3A and DNMT3L bind H3 tails (Fig. 1*B*, V. or VI.) (11, 12, 13, 14, 15, 16). We initially challenged this notion by assessing whether DNMT3A–p53 or DNMT3A–TDG heterotetramers can bind H3K4me0 peptide. The increase in anisotropy observed following the addition of H3K4me0 peptide to FAM-labeled DNA bound by DNMT3A–p53 or –TDG complexes shows that binding of H3 tails and these regulatory proteins is not mutually exclusive (Fig. 2*A*) and that DNMT3A–H3 tail-p53 or -TDG complexes remain bound to DNA. Based on findings from distinct studies, p53 or TDG appear to display a stronger affinity for DNMT3A relative to H3 peptides (10, 13, 14, 19). Consistent with this notion, we found that inhibition of DNMT3A activity by p53 or TDG in DNMT3A–H3 tail-p53 or –TDG complexes is dominant in the presence of H3K4me0 or H3K4me3 peptide (Fig. 2*B*) in reactions at equilibrium. Moreover, the dominant modulatory effect on DNMT3A activity by p53 or TDG persists in actively methylating DNMT3A–H3K4me0 (Fig. 2, *C*–*D*) or -H3K4me3 (Fig. 2, *E*–*F*) complexes. The results of transient reactions (Fig. 2, *C*–*F*) better model the cellular interactions of these epigenetic mechanisms and show that binding of H3 to its allosteric site does not induce conformational changes of DNMT3A that hinder the modulation of DNMT3A activity by p53 or TDG. Furthermore, equimolar concentrations of p53 and TDG relative to DNMT3A (150 nM tetramer) were used in all reactions (Fig. 2, *B*–*F*), suggesting that the dominant modulation of DNMT3A activity by p53 and TDG over H3 peptides was not because of stoichiometric differences. We propose that DNMT3A simultaneously accommodates H3 tails and p53 or TDG to form complexes that are similar to the DNMT3A–DNMT3L–H3 tail co-crystal structure (Fig. 1*A*) and in which the primary role of p53 or TDG is to modulate DNMT3A activity.

The spatial relationship between nucleosomes and nucleosome-interacting proteins (intranucleosomal or internucleosomal, Fig. 3*A*) provides a greater understanding of the interactions associated with readers, writers, and erasers within chromatin. Compared with the interactions of histone-modifying enzymes and tails within a single nucleosome, the interactions of histone-modifying enzymes and histone tails on adjacent nucleosomes have proven more challenging to study because of the difficulty in generating suitable substrates that distinguish the two types of interactions. However, some studies have successfully characterized these two types of interactions in histone-modifying enzymes (17, 18). Structural analysis of DNMT3A and nucleosomes suggests steric hindrance from the comparable sizes (length, diameter, and height) of the DNMT3A homotetramer, and nucleosomes may pose a challenge for DNMT3A to act on intranucleosomal DNA (Fig. S3) (30, 31). We sought to explore the spatial relationship between DNMT3A and nucleosome substrates (intranucleosomal or internucleosomal, Fig. 3*A*) in more detail before assessing the ability of p53 or TDG to modulate the enzymatic activity of DNMT3A on nucleosomes. We initially assessed the extent to which the N-terminus of DNMT3A contributes to DNMT3A–mononucleosome interactions by comparing the enzymatic activity of full length DNMT3A (residues 1–912) on nucleosomes to that of the catalytic domain of DNMT3A (residues 634–912) and the prokaryotic DNA methyltransferase M. SssI. We show the N-terminal domains of DNMT3A (ADD and PWWP) enhance the enzymatic activity of DNMT3A on nucleosomes, likely by retaining DNMT3A on nucleosome substrates (Fig. S1) (10, 12, 22). The H3K4me0 peptide allosterically activates the enzymatic activity of DNMT3A on a variety of oligonucleotide substrates (Fig. 2) (10, 19). We therefore examined whether extrinsic H3K4me0 peptide (non-nucleosomal) stimulates the activity of DNMT3A on mononucleosomal DNA or if binding of DNMT3A to intrinsic (nucleosomal) H3 tails perturbs the activation of DNMT3A by H3K4me0 peptide (Fig. 3*B*). We show binding of histone tails within nucleosomes and the short H3 peptide to DNMT3A are mutually exclusive, and nucleosome bound DNMT3A is not accessible to H3 peptides (Fig. 3, *B*–*C*). To further distinguish between intranucleosomal or internucleosomal interactions (Fig. 3*A*), we then assessed the interactions of DNMT3A bound to nucleosomes with extrinsic DNA (non-nucleosomal). We show DNMT3A–nucleosome complexes can bind (Fig. 3*F*) and act on distinct extrinsic DNA substrates (Fig. 3, *D*–*E*) as the changes in DNMT3A activity observed by the addition of pCpG^L^ or Poly dI-dC (Fig. 3, *D*–*E*) may only be achieved by DNMT3A employing an internucleosomal mechanism (Fig. 3*A*, I.). Taken together, nucleosome bound DNMT3A is not limited to methylating intranucleosomal DNA and can act on internucleosomal substrates (Fig. 3*A*, I.). These results suggest that cues provided by particular histone modifications may result in DNMT3A-mediated methylation of nucleosomal DNA in a particular region, encompassing DNA not directly associated with the nucleosome to which the enzyme is bound.

Functional characterization of DNMT3A–H3 tail-regulatory protein complexes indicates that regulatory proteins play a dominant role over histone H3 peptide in the regulation of DNMT3A activity (Fig. 2). To better approximate the simultaneous modulation of DNMT3A activity within cells, we then assessed the relative role (dominant or passive) of histone H3 tails and regulatory proteins using human-derived mononucleosome or polynucleosome substrates. Like histone-modifying enzymes, which are differentially regulated on nucleosomes compared with peptide substrates and whose enzymatic activity is influenced by nucleosome number (mononucleosome or polynucleosome), we found that a higher concentration of polynucleosomes compared with mononucleosomes is required for DNMT3A to reach comparable maximal velocities on either substrate (Fig. S2). This may result from the greater challenge for DNMT3A to access DNA in polynucleosome because of the structural complexity of this substrate. We show that equimolar concentrations of p53 or TDG relative to DNMT3A (150 nM tetramer) sufficiently modulate the enzymatic activity of DNMT3A in equilibrium (Fig. 4*A*) or transient (Fig. 4, *B*–*C*) reactions with mononucleosome substrates. In contrast, we found that the activity of DNMT3A on polynucleosomes is modulated by equimolar concentrations of p53 or TDG (1:1 relative to 150 nM tetramer DNMT3A) only in equilibrium reactions (Fig. 4*A*) and that transient reactions require the addition of excess p53 (500 nM tetramer) or TDG (500 nM dimer) (Fig. 4, *D*–*E*). P53 and TDG complexes with DNMT3A are more stable than DNMT3A–nucleosome complexes (Fig. S2) (13, 14). Therefore, the results of reactions with polynucleosomes in which equimolar concentration of p53 or TDG to DNMT3A modulate the enzymatic activity of DNMT3A could be attributed to the thermodynamic regulation of the interactions between DNMT3A, regulatory proteins, and mononucleosomes (Fig. 4, *A*–*C*) or polynucleosomes (Fig. 4*A*). Interactions between the N-terminal domains of DNMT3A (ADD and PWWP) and histone H3 tails not only promote the retention of DNMT3A to polynucleosomes (Fig. S1), but present additional interactions that may pose a challenge for allosteric regulators of DNMT3A to access DNMT3A under catalytic conditions. We show that an excess concentration of p53 or TDG to DNMT3A is necessary to overcome this challenge and modulate the enzymatic activity of DNMT3A (Fig. 4, *D*–*E*), which is likely an appropriate representation of what occurs within cells as the expression of p53 or TDG is highly dynamic (32, 33). We propose that the histone code represents a network of specific modifications which creates a focal point for recruitment of DNMT3A (Fig. 1*B*). Thus, DNMT3A–H3 tail interactions in DNMT3A–H3 tail-p53 or -TDG complexes increase the local concentration of DNMT3A at specific regions, while the primary role of p53 or TDG is to modulate DNMT3A activity (Fig. 1*B*, II. and III.).

Genome-wide epigenetic profiling has provided unprecedented information about the links between specific histone marks and DNA methylation (4, 5, 6, 7, 8, 9). However, these associations do not consider the role of regulatory proteins and their dynamics with epigenetic enzymes in transcriptional regulation, though many aspects of epigenetic transcriptional regulation stem from the direct modulation of epigenetic enzymes by protein partners (13, 14, 15, 16). Most histone modifications do not work in isolation but rather form a histone code, with the combination of all modifications influencing the recognition and activity of readers, writers, or erasers (34). When the role of proteins that modulate readers, writers, or erasers is considered in addition to cues presented by the histone code, the complexity of the dynamics associated with epigenetic transcriptional regulation becomes clear. To date, biochemical work aiming to explore the interactions between DNMT3A, regulatory proteins, and histone tails have solely focused on DNMT3L (10, 11, 12). We provide insights into two important cancer-related proteins that directly (DNA methylation) and indirectly (histone modifications) affect key epigenetic mechanisms of how the simultaneous binding of regulatory proteins and H3 tails at distinct surfaces may affect enzymatic activity, thereby providing insights into the interactions that contribute to mammalian DNA methylation (13, 14, 15, 16). The expression of DNMT3L is limited to germ cells and early developmental stages, whereas the expression of p53 and TDG, like DNMT3A, is not only highly dynamic, but p53, TDG, and DNMT3A are active in a wide range of cellular contexts (15, 16, 32, 33, 35, 36, 37, 38, 39). Furthermore, disruptions to the cellular activity of DNMT3A, p53, or TDG have been implicated in human cancers such as AML, and we have shown in previous work that clinically identified mutations in p53 disrupt the interactions of DNMT3A with additional partner proteins (3, 40, 41, 42). Future studies will be required to explore whether mutations in allosteric regulators of DNMT3A, like p53 and TDG, disrupt the interactions of DNMT3A with histone H3 tails and lead to differential functional outcomes. Thus, the findings in this study expand our understanding of the interactions associated with the modulation of readers and writers of epigenetic marks by regulatory proteins with broad biological implications.

## Experimental procedures

### Expression constructs

The following plasmids were used for expression of recombinant human proteins: pET28a-hDNMT3ACopt for DNMT3A full length (43), pET28a-hDNMT3A_catalytic_domain for DNMT3A catalytic domain (Δ1–611) pET28a-hDNMT3A_catalytic_domain (Δ1–611) (26), pET15b-human p53 (1–393) for p53 (44), and pET28a-hTDG for TDG (45).

### Protein expression

DNMT3A full length and catalytic domain, p53, and TDG were expressed in NiCo21(DE3) Competent *E. coli* cells (New England Biolabs). Cells were grown in LB media at 37 °C to an A_600 nm_ of 0.9 (DNMT3A full length), 0.7 (DNMT3A catalytic domain), 0.6 (p53), and 0.8 (TDG). Protein expression was induced by the addition of 1 mM isopropyl-β-D-thiogalactopyranoside (GoldBio) after lowering the temperature to 28 °C. Induction times were 5 h for DNMT3A full length and catalytic domain and 16 h for p53. Cell pellets were harvested by centrifugation at 5000*g* for 15 min and stored at −80 °C.

### Protein purification

Cell pellets from 1 l of bacterial culture were resuspended in 30 ml lysis buffer (50 mM HEPES pH 7.8, 500 mM NaCl, 50 mM imidazole, 10% glycerol, and 1 mM PMSF) and lysed by sonication. Following sonication, lysates were centrifuged at 11,000*g* for 1 h, and the supernatant was retained for affinity chromatography. Recombinant proteins were purified using ÄKTA Fast Protein Liquid Chromatography system (GE healthcare) containing a 5 ml HisTrap HP nickel-charged IMAC column (GE healthcare). Columns were equilibrated with 50 ml of loading buffer (50 mM HEPES pH 7.8, 500 mM NaCl, 50 mM imidazole, 10% glycerol). After flowing the supernatant through the column, resins were washed using 47.5 ml of wash buffer (50 mM HEPES pH 7.8, 500 mM NaCl, 75 mM imidazole, 10% glycerol). 0.5 ml fractions were eluted with increasing amounts of imidazole (50 mM HEPES pH 7.8, 500 mM NaCl, 75–500 mM imidazole, and 10% glycerol) over 15 ml. The fractions containing the proteins of interest was desalted and concentrated into storage buffer (50 mM Tris-Cl, 200 mM NaCl, 1 mM EDTA, 20% (v/v) glycerol, pH 7.8, with 0.5 mM DTT) using a 0.5 ml Centrifugal Filter (Millipore 10K device) supplied by Millipore and were stored at −80 °C for later use. Protein concentrations were determined using 280 nm extinction coefficients (142,010 M^−1^ cm^−1^ for full length DNMT3A, 38,180 M^−1^ cm^−1^ for the catalytic domain of DNMT3A, 36,035 M^−1^ cm^−1^ for p53 and 33,725 M^−1^ cm^−1^ for TDG) and reflect the oligomeric state in all experimental conditions (nM of tetramers for full length DNMT3A, the catalytic domain of DNMT3A and p53; nM of dimers for TDG). A summary gel of the purified recombinant proteins used in this study is in Fig. S4.

### Methylation assays

Radiochemical assays were carried out to measure the ability of DNMT3A to incorporate tritiated methyl groups transferred from cofactor AdoMet onto distinct DNA substrates and under varying experimental conditions. In this study, DNMT3A refers to the full-length protein (912 amino acids), unless noted otherwise. Reactions were carried out at 37 °C in a buffer consisting of 50 mM KH_2_PO_4_/K_2_HPO_4_, 1 mM EDTA, 1 mM DTT, 0.2 mg/ml BSA, 20 mM NaCl with saturating AdoMet (15 μM) at pH 7.8. For the radiochemical assays, 50 μM ([^3^H] methyl-labeled: unlabeled, 1:10) AdoMet stocks were made using 32 mM unlabeled AdoMet (NEB) and [^3^H] methyl-labeled AdoMet (80 Ci/mmol) supplied by PerkinElmer in 10 mM H_2_S O_4_. 15 μl aliquots were taken from a larger reaction, quenched by mixing with 0.1% SDS (1:1), and spotted onto Hybond-XL membranes (GE healthcare). Samples were then washed, dried, and counted using a Beckman LS 6000 liquid scintillation Counter as previously established (46).

### Methylation assays with H3K4me0 or H3K4me3 peptides in combination with p53 and TDG

Synthetic peptides (N-ARTKQTARKSTGGKAPRKQLA-C) derived from human Histone H3.1 were supplied by Active Motif (47). In equilibrium reactions containing H3K4me0 or H3K4me3 peptides, 4 μM of either peptide was preincubated with DNMT3A and individual regulatory proteins (p53 tetramers or TDG dimers, 1:1 at 150 nM) in reaction buffer with AdoMet for 1 h at 37 °C before initiating the reaction by the addition of saturating substrate DNA (5 μM poly dI-dC). In transient reactions, DNMT3A was preincubated with individual peptides (4 μM of H3K4me0 or H3K4me3) in reaction buffer with AdoMet for 1 h at 37 °C, and reactions were initiated by the addition of DNA (5 μM poly dI-dC) and allowed to carry out catalysis for 30 min before the addition of p53 or TDG.

### Methylation assays with human mononucleosomal or polynucleosomal DNA

Unmodified recombinant human mononucleosomes consisting of two molecules of each of the four core histones (H2A, H2B, H3.1, and H4) bound by the Widom 601 positioning sequence (147 base pairs and 13 CpG sites) were supplied by Active Motif (48). Human polynucleosomes were generated from HeLa cell nuclear extracts subjected to micrococcal nuclease digestion. Purified HeLa polynucleosomes consisting of predominantly trimers of the histone octamer (two each of the four core histones, H2A, H2B, H3, and H4) wrapped by 147 base pairs of human genomic DNA were supplied by EpiCypher (49). The concentrations of mononucleosomes or polynucleosomes were determined by the absorbance at 280 nm, using the molecular weight of histone octamer (108 kDa). Equilibrium reactions consisting of reaction buffer with AdoMet, mononucleosomes or polynucleosomes (1 μM), and individual regulatory proteins (p53 or TDG, 1:1 at 150 nM) were initiated by the addition of DNMT3A. In transient reactions, p53 or TDG (1:1 at 150 nM) were added to actively catalyzing DNMT3A on mononucleosomes or polynucleosomes (1 μM) after 1 h. To assess the accessibility of exogenous peptides to DNMT3A acting on mononucleosomes, additional equilibrium and transient experiments were performed. Equilibrium reactions consisting of reaction buffer with AdoMet, mononucleosomes (1 μM), and increasing levels of H3K4me0 were initiated by the addition of DNMT3A (150 nM). In transient reactions, increasing levels of H3K4me0 were added to actively catalyzing DNMT3A on mononucleosomes (1 μM) after 1 h. The accessibility of exogenous DNA was also assessed by the addition of excess Poly dI-dC or pCpG^L^ (20X) to actively catalyzing DNMT3A (150 nM) on mononucleosomes (1 μM). The concentrations of Poly dI-dC or pCpG^L^ are given in base pairs and were determined by the absorbance at 260 nm using the following molar absorptive coefficients: 6.9 mM^−1^ cm^−1^ for Poly dI-dC and 6.6 mM^−1^ cm^−1^ for pCpG^L^.

### Fluorescence anisotropy

Fluorescence anisotropy measurements were obtained using a Horiba Fluoromax ﬂuorescence spectrophotometer equipped with excitation and emission polarizers (excitation: 485 nm, emission 520 nm). The DNA substrate (Gcbox30) consisted of a fluorescein (6-FAM) label on the 5′ end of the top strand of the duplex (5′/6-FAM/TGGATATCTAGGGGCGCTATGATATCT-3′) was supplied by Integrated DNA Technologies. The recognition site for DNMT3A is underlined. In DNA binding experiments of homotetrameric or heterotetrameric complexes, unlabeled H3K4me0 peptide was titrated to preformed DNMT3A or DNMT3A-regulatory protein (p53 and TDG) complexes (1 μM). Anisotropy values were obtained following the addition of unlabeled H3K4me0. To assess the ability of nucleosome-bound DNMT3A to bind non-nucleosomal DNA, increasing concentrations of preformed DNMT3A–mononucleosome complexes (or DNMT3A only) were added to 15 nM Gcbox30. For peptide binding experiments, H3K4me0 peptides were labeled with FAM–NHS on the N-terminus. Unlabeled mononucleosomes were then added to FAM-labeled H3K4me0 (2 μM) bound by DNMT3A (150 nM). Anisotropy values were obtained following a 5-min incubation at room temperature for all experiments.

## Data availability

All data that support the findings of this study are contained within the manuscript and are available from the corresponding author upon reasonable request.

## Conflict of interest

The authors declare that they have no conflicts of interest with the contents of this article.
