# Supplementary material for: p53 and TDG are dominant in regulating the activity of the human de novo DNA methyltransferase DNMT3A on nucleosomes
Source: J Biol Chem. 2020 Nov 24;296:100058. doi: 10.1074/jbc.RA120.016125 (PMC7948466; doi:10.1074/jbc.RA120.016125)
Supplement: Figures S1–S6 [file mmc1.pdf]

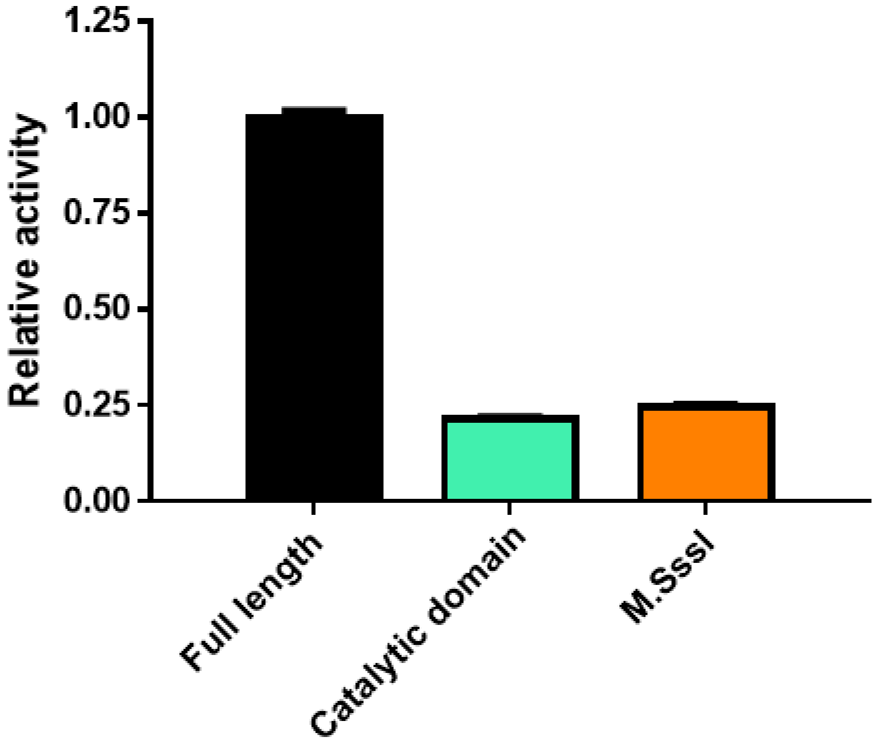

Figure S1. Interactions between the N-terminus of DNMT3A and nucleosomes increase catalytic activity on nucleosomal DNA. Compared to full length DNMT3A (150 nM) (■), the catalytic domain of DNMT3A (150 nM) (■) and the bacterial CpG DNA methyltransferase M. SssI (0.2 units/µL) (■) are less catalytically active on mononucleosomal DNA (1 µM) as a substrate. Data were normalized to the DNA methylation activity observed in full length DNMT3A and reflect the results of 2 independent experiments.

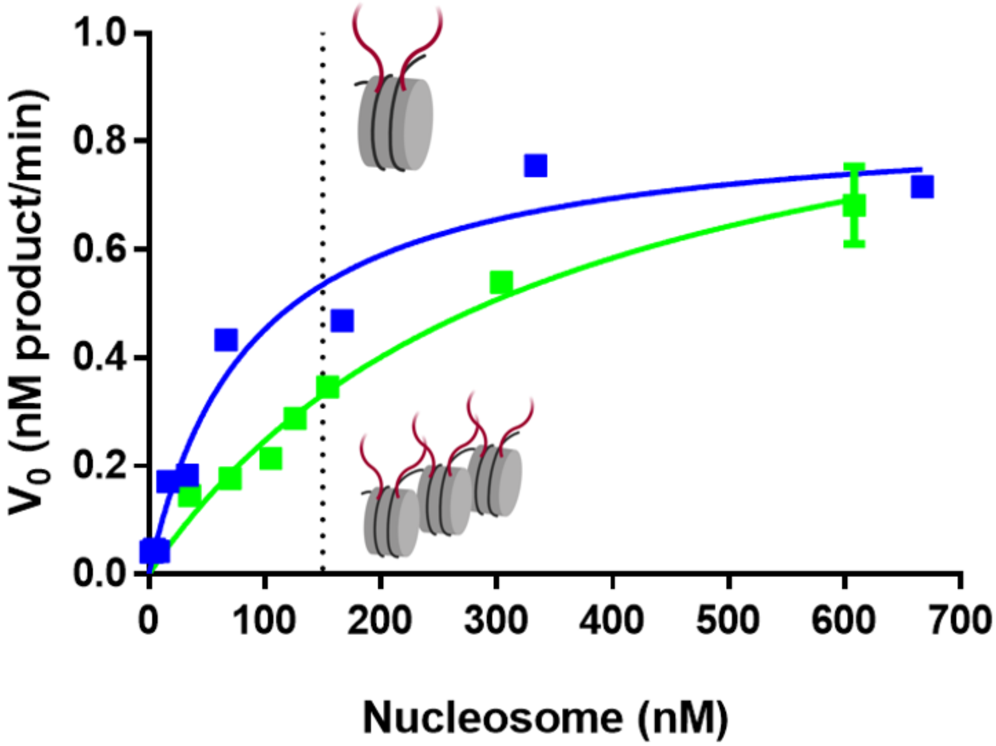

Figure S2. Binding curve of DNMT3A with mono- or poly-nucleosomal DNA as a substrate. Titration of (■) mono- or (■) poly-nucleosomes to DNMT3A (150 nM tetramer). Dashed line indicates equal concentration of mono- or poly-nucleosomes (150 nM) relative to DNMT3A. Data are representative of reactions carried out for 60 minutes and reflect the results of 2 independent experiments.

A.

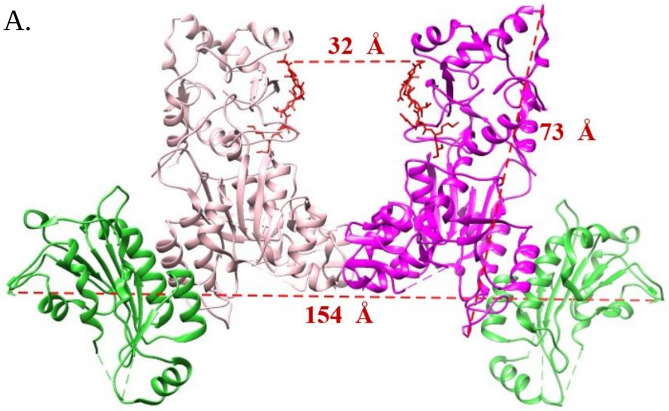

B.

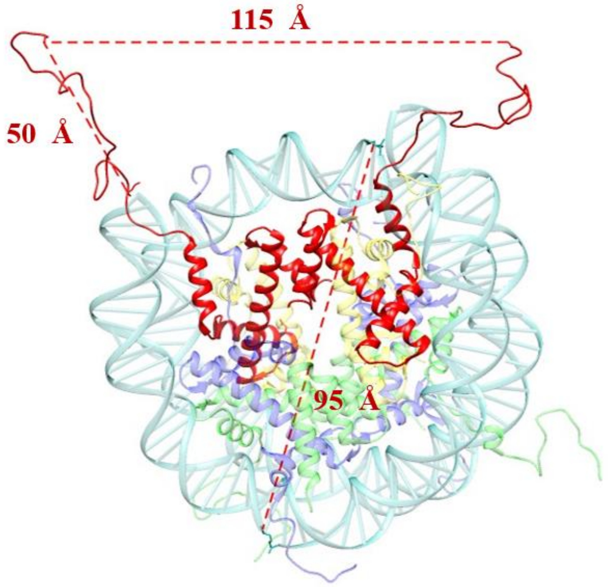

C.

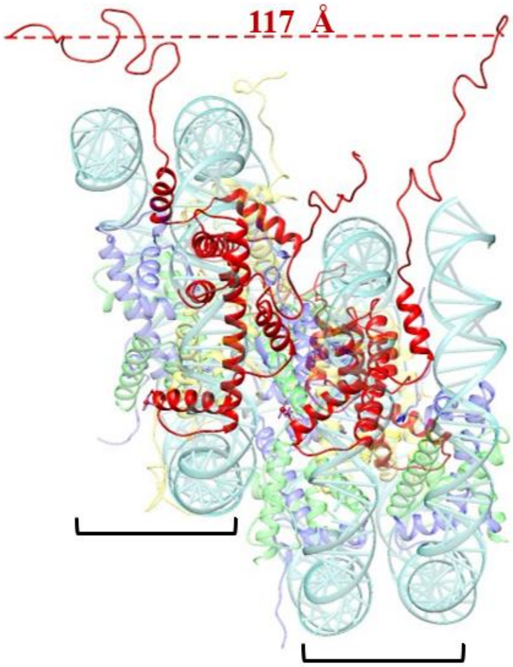

Figure S3. Distances in DNMT3A-Nucleosome interactions. (A.) Distances on a DNMT3A-DNMT3L heterotetramer in complex with Histone H3 N-terminal peptide (adapted from PDB 4U7T) (10). Distance between (B.) intra and (C.) inter -nucleosomal H3 N-terminal tails as well as nucleosome diameter and height of H3 N-terminal tail (B.). To generate a model of a di-nucleosome containing H3 N-terminal tails (B.), H3 (chain A) from PDB 1KX5 (30) was superimposed to H3 (chains A, E, K, O; Δ1–40) from PDB 5GSE (RMSD < 0.4 Å) (31). Modelling and distances were generated in UCSF Chimera.

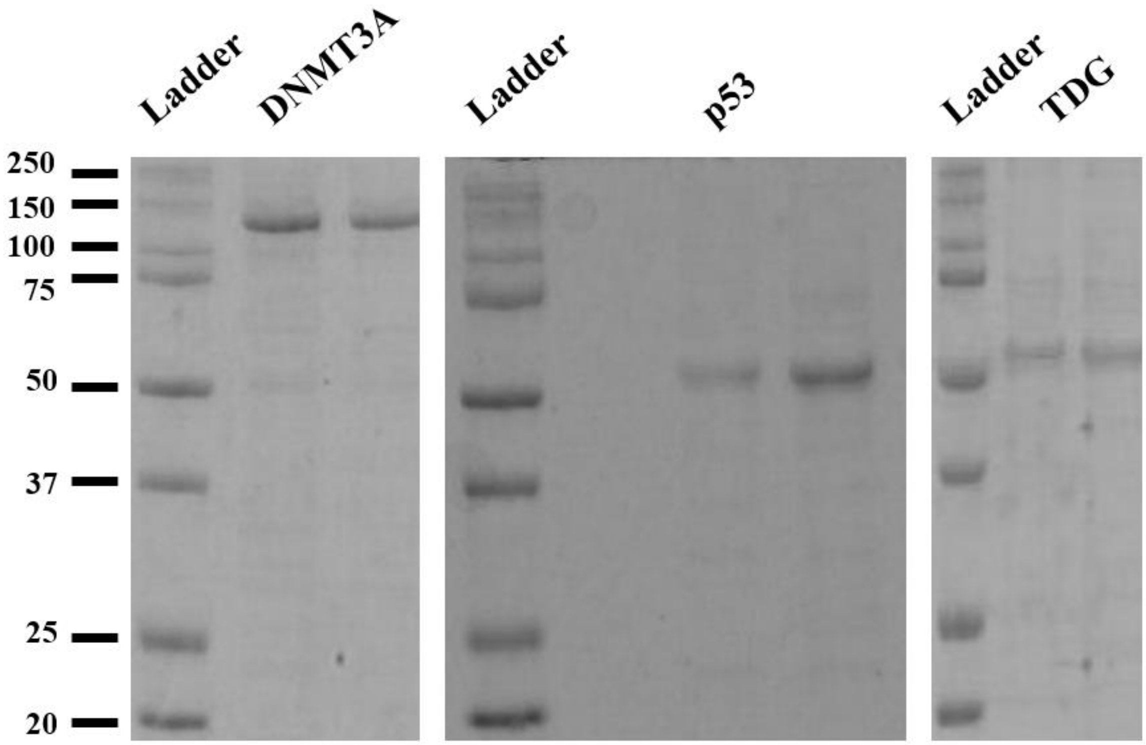

Figure S4. 10% SDS-PAGE gel of the purified proteins. Bio-rad Precision Plus Protein Dual Color was used as a size standard. The estimated molecular weight of purified proteins are as follows: 130 kDa for full length DNMT3A, 53 kDa for p53 and 55 kDa for TDG. Two lanes were loaded from pooled fractions of each purified protein.

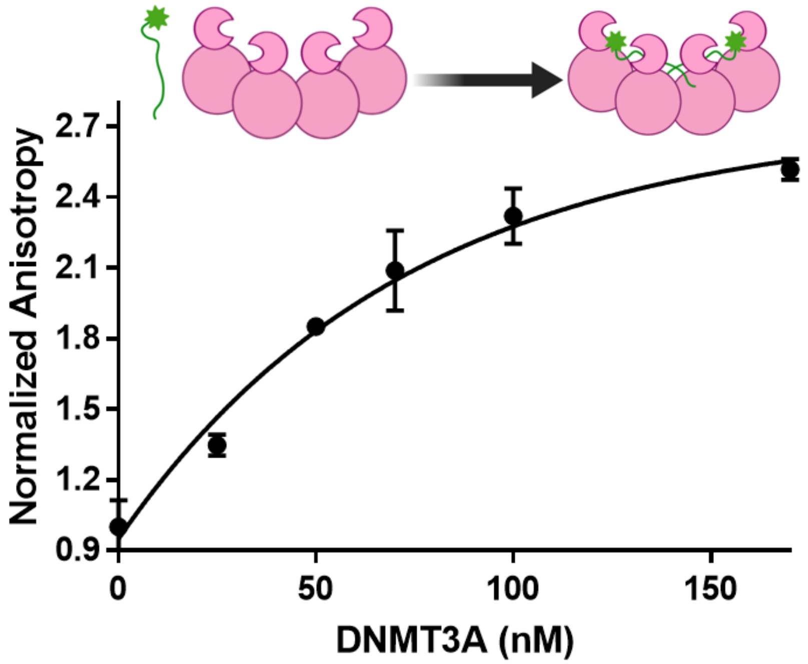

Figure S5. Binding curve DNMT3A to FAM-labeled H3K4me0 peptides. Increasing concentrations of full-length DNMT3A (0-170 nM) leads to a concomitant increase in the fluorescence anisotropy of FAM-labelled H3K4me0 (2 µM) (residues 1-21). Data are normalized to fluorescence anisotropy values in the absence of DNMT3A.

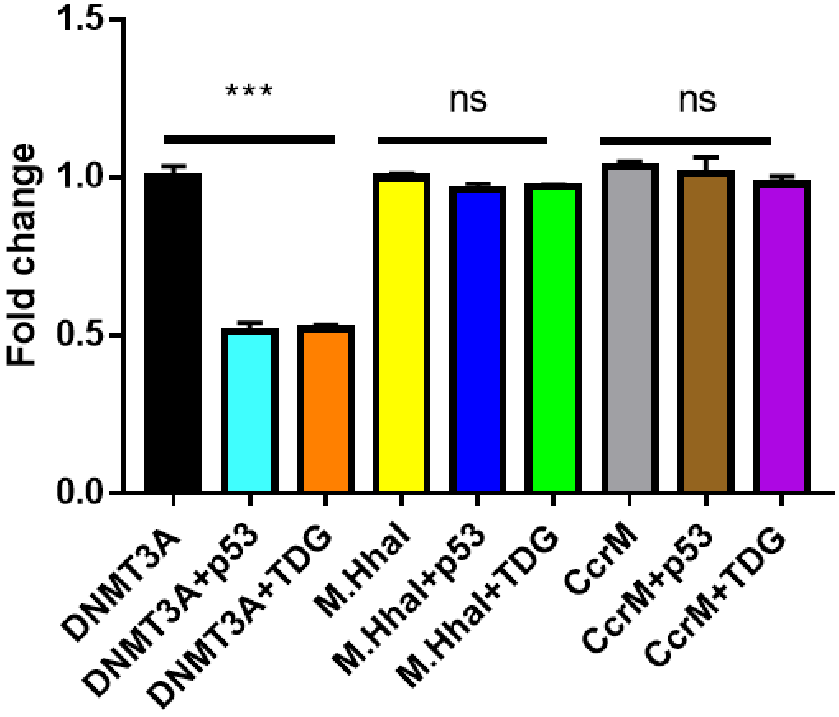

Figure S6. P53 and TDG specifically inhibit the DNA methylation activity of DNMT3A. The activity of bacterial CpG methyltransferase M.HhaI and adenine methyltransferase CcrM was assessed in the presence of p53 or TDG to assess the specificity of the modulation of DNMT3A activity by p53 and TDG. In all reactions, DNA methyltransferase (DNMT3A, M.HhaI and CcrM) were at 150 nM while p53 and TDG were at 500 nM. For co-incubations with p53 or TDG, proteins were placed at 37 °C for 1 hour prior to the addition of substrate DNA (Poly dI-dC at 5 μM for M.HhaI and DNMT3A; dsDNA 29 mer 5' TCACTGTACTCTGACTCGCCTGACATGAC 3' for CcrM. Data were normalized to the DNA methylation activity observed in the absence of p53 or TDG and are representative of reactions carried out for 1 hour. Data reflect the mean ± S.D. of 3 experiments; one-way analysis of variance was used to compare the values of reactions with p53 or TDG to those with DNA methyltransferases only; ***, $p < 0.001$; ns, $p > 0.05$. Data were normalized to the DNA methylation activity observed in the absence of p53 or TDG and are representative of reactions carried out for 1 hour. Data reflect the mean ± S.D. of 3 experiments; one-way analysis of variance was used to compare the values of reactions with p53 or TDG to those with DNA methyltransferases only; ***, $p < 0.001$; ns, $p > 0.05$.
